# Universal conventional and real-time PCR diagnosis tools for *Sarcoptes scabiei*

**DOI:** 10.1186/s13071-015-1204-8

**Published:** 2015-11-14

**Authors:** Samer Angelone-Alasaad, AnnaRita Molinar Min, Mario Pasquetti, Abdulaziz N. Alagaili, Stefano D’Amelio, Federica Berrilli, Vincent Obanda, Mohamed A. Gebely, Ramón C. Soriguer, Luca Rossi

**Affiliations:** Dipartimento di Scienze Veterinarie, Largo Paolo Braccini 2, 10095 Grugliasco, Italy; Institute of Evolutionary Biology and Environmental Studies (IEU), University of Zürich, Winterthurerstrasse 190, 8057 Zürich, Switzerland; Department of Zoology, College of Science, King Saud University, Riyadh, Saudi Arabia; Department of Public Health and Infectious Diseases, University of Rome La Sapienza, Rome, Italy; Department of Experimental Medicine and Surgery, University of Rome Tor Vergata, Rome, Italy; Department of Veterinary Services, Kenya Wildlife Service, Nairobi, Kenya; Department of Parasitology and Animal Diseases, Veterinary Research Division, National Research Center, Dokki, Giza Egypt; Estación Biológica de Doñana, Consejo Superior de Investigaciones Científicas (CSIC), Avda. Américo Vespucio s/n, 41092 Sevilla, Spain

**Keywords:** Sarcoptic mange, Scabies, Host species, Mitochondrial 16S rDNA, Conventional end-point PCR, TaqMan real time PCR, *Sarcoptes* WMN

## Abstract

**Background:**

The mite *Sarcoptes scabiei* has a known host-range of over 100 mammal species including humans. One of the prime objectives of the *Sarcoptes*-World Molecular Network (WMN) is to design and develop universal *Sarcoptes* PCR-based diagnosis methods.

**Methods:**

We describe here for the first time two universal mitochondrial-based diagnosis methods: (i) conventional end-point PCR and (ii) TaqMan real-time PCR. The design of both of these universal diagnosis methods was based on *Sarcoptes* samples collected from 23 host species in 14 countries.

**Results:**

These methods, based on skin scrapings, were successfully used to etiologically confirm the diagnosis of different clinical degrees of sarcoptic mange in 48 animals belonging to six species. These universal PCR-based diagnosis methods are highly specific, technically sensitive and simple, and are based on the amplification of 135 bp from the Mitochondrial 16S rDNA. The method based on TaqMan real-time qPCR was more sensitive than the conventional end-point PCR.

**Conclusions:**

Two universal PCR-based diagnosis methods for *S. scabiei* were successfully designed and applied; one based on conventional end-point PCR and the other on TaqMan real-time PCR. We recommend further testing and the application of these new universal methods worldwide.

## Background

The mite *Sarcoptes scabiei* is the origin of sarcoptic mange in pets, livestock and wild animals, and of scabies in humans. Its very wide host-range (host-derived *Sarcoptes*) includes over 100 mammalian species belonging to 27 families and 10 orders [1, 2]. As the agent of mange in both wild and domestic animals it can cause significant economic losses given its ability to negatively affect animal production and welfare, and lead to increased mortality. In humans, this mite triggers outbreaks of scabies, a globally distributed emerging/re-emerging infectious disease that is especially prevalent in developing countries [3]. It is calculated that, worldwide, 300 million people are infested with scabies, although this figure probably underestimates the true situation given that this infection is often not reported in humans [4].

Regardless of the methods used, the diagnosis of mange or scabies continues to be a challenge [5, 6]. Some of the numerous techniques currently employed to detect this infection include (a) clinical diagnosis (via clinical signs and histo-pathological examination of bioptic samples); (b) microscopy diagnosis (identification of mites, eggs, eggshell fragments and/or mite faecal pellets from skin scrapings); (c) dermatoscopical diagnosis (epiluminescence microscopy and high-resolution videodermatoscopy); (d) intradermal skin tests; (e) antibody detection; (f) antigen detection; (g) PCR-based diagnosis; and (h) mange-detector dogs [6, 7].

Studies carried out using molecular markers generally aim to evaluate how *S. scabiei* population genetics affects hosts of different geographical origin [8].

Scabies control in humans, eradication programs of sarcoptic mange in farm animals [9, 10] and studies of the epidemiology and pathology of this condition in a range of animal species (including vulnerable wildlife) [11] would clearly benefit from improved methods that are more sensitive to infection by *Sarcoptes* mites. The aim of this paper was to report the design and application of two new universal diagnosis methods of *Sarcoptes scabiei* based on traditional end-point PCR and real-time TaqMan PCR following the recommendations made by the *Sarcoptes*-World Molecular Network [1].

## Methods

### Sample collection

A total of 39 *Sarcoptes* mites were individually collected using different isolation methods [12] from the skin of 23 host species from 14 countries (Table 1). To test the new tools, we also collected 48 skin scrapings from the following animals (samples were collected from dead animals for post-mortem diagnostic purposes): (a) two healthy unexposed roe deer (*Capreolus capreolus*); (b) four healthy unexposed badgers (*Meles meles*); (c) five red foxes (*Vulpes vulpes*) with different degrees of mange; (d) 14 northern chamois (*Rupicapra rupicapra*) with different degrees of mange; (e) a mange-affected red deer (*Cervus elaphus*); and (f) 22 Iberian ibex (*Capra pyrenaica*), of which three were healthy and unexposed, and 19 mangy (Table 2). All skin samples from mangy animals were microscopically confirmed to be *S. scabiei* positive.

**Table 1:** *Sarcoptes scabiei* samples used in the design of the primers and the post-optimization evaluation of the universal PCR-based diagnosis method

Geographical origin	Host species	No. of samples
Korea	Human (*Homo sapiens sapiens*)	1
Brazil	Human (*Homo sapiens sapiens*)	1
France	Human (*Homo sapiens sapiens*)	1
Italy	Northern chamois (*R. rupicapra*)	2
Spain	Southern chamois (*R. pyrenaica*)	1
Spain	Spanish ibex (*Capra pyrenaica*)	2
Italy	Alpine ibex (*Capra ibex*)	1
Italy	Red fox (*Vulpes vulpes*)	2
Spain	Red fox (*Vulpes vulpes*)	1
Italy	Wild boar (*Sus scrofa*)	3
Spain	Rabbit (*Oryctolagus cuniculus*)	3
Germany	Raccoon (*Procyon lotor*)	3
Tanzania	Wildebeest (*Connochaetes taurinus*)	1
Japan	Raccoon dog (*Nyctereutes procyonoides*)	1
West Indies	Dog (*Canis lupus familiaris*)	1
Argentine	Capybara (*Hydrochoerus hydrochaeris*)	1
Italy	Bovine (*Bos taurus*)	1
Italy	Red deer (*Cervus elaphus*)	1
Spain	Red deer (*Cervus elaphus*)	1
Italy	Stone marten (*Martes foina*)	1
Switzerland	Eurasian lynx (*Lynx lynx*)	1
Italy	Mouflon (*Ovis aries musimon*)	1
Spain	Grey wolf (*Canis lupus*)	1
Egypt	Sheep (*Ovis aries*)	1
Kenya	Thomson’s gazelle (*Eudorcas thomsonii*)	1
Kenya	Lion (*Panthera leo*)	1
Kenya	Cheetah (*Acinonyx jubatus*)	1
Kenya	Dog (*Canis lupus familiaris*)	1
Kenya	Reticulated giraffe (*Giraffa camelopardalis reticulata*)	1
Tunisia	Dromedary camel (*Camelus dromedarius*)	1

**Table 2:** Skin-scraping samples used in the evaluation of the universal PCR-based diagnosis method

Geographical origin	Host species	No. of mangy samples	No. of healthy samples
Italy	Roe deer (*Capreolus capreolus*)	0	2
Italy	Badgers (*Meles meles*)	0	4
Italy	Red foxes (*Vulpes vulpes*)	5	0
Italy	Northern chamois (*Rupicapra rupicapra*)	14	0
Italy	Red deer (*Cervus elaphus*)	1	0
Spain	Iberian ibex (*Capra pyrenaica*)	19	3

### DNA extraction

The HotSHOT Plus ThermalSHOCK technique [13] and NucleoSpin Tissue kit procedure (Macherey-Nagel, Düren, Germany) [14] were employed to extract genomic DNA from all individual mites. The success rate of DNA extraction from parasites was about 70 % depending on the method used (live or dead mites) and the type of preservation (frozen or in ethanol) [14, 15]. DNA was extracted from the skin scrapings using the two above-mentioned methods with minor modifications (e.g. we used twice as much reagent as we used to extract genomic DNA).

### Amplification and sequencing of a fragment from the Mitochondrial 16S rDNA

A fragment from the Mitochondrial 16S rDNA (407 bp) was amplified by PCR using primers 16S-F and 16S-R as reported previously [10] in a 2720 thermal cycler (Applied Biosystems, Foster City, California). The amplicons were examined on 1.5 % agarose gel stained with ethidium bromide for DNA visualization under UV light. The purified PCR products were directly cycle-sequenced from both directions on ABIPRISM 310 Genetic Analyser (Applied Biosystems, Foster City, California) using the BigDye Terminator Cycle Sequencing Kit 1.1 (Applied Biosystems, Foster City, California). Individual mite consensus sequences were manually trimmed of primer sequences, aligned, compared and edited using BioEdit v7.0.9.0 [16].

### Universal primer design

Based on the comparison of the obtained sequences, we used Primer 3 (v. 0.4.0) [17] to design a set of universal primers for the amplification of *S. scabiei* with an estimated size of 135 bp. The forward primer was SSUDF (5′-GGGTCTTTTTGTCTTGGAATAAA-3′) and reverse primer SSUDR (5′-CTAAGGTAGCGAAATCATTAGC-3′).

### The end-point PCR universal diagnosis method protocol

The final protocol for the diagnosis of *S. scabiei* after adjusting the PCR mixture and the annealing temperature consisted of a total volume of 30 μL PCR mixture composed of 3 μL of single *Sarcoptes* DNA, 200 μM of each dNTP, 0.1 μM of each primer, 3 μL of 10X PCR buffer (100 mMTris–HCl, pH 8.3 and 500mMKCl), 1.5 mM MgCl_2_ and 0.3 μL (1.5 U/reaction) Hot-Start Taq DNA polymerase (Qiagen, Milano, Italy). Samples were subjected to the following thermal profile for amplification in a 2720 thermal cycler (Applied Biosystems, Foster City, California): 15 min at 95 °C (initial denaturing), followed by 35 cycles consisting of three steps of 30 s at 94 °C (denaturation), 45 s at 53 °C (annealing) and 1.5 min at 72 °C (extension), before a final elongation of 7 min at 72 °C. The amplicons were examined on 2 % agarose gel and stained with ethidium bromide for DNA visualization under UV light.

### The TaqMan real-time PCR universal diagnosis method protocol

The TaqMan real-time PCR probe relies on the 5′–3′ exonuclease activity of Taq polymerase, which cleaves a dual-labelled probe in the hybridization phase to the complementary target sequence and fluorophore-based detection [18]‬. The resulting fluorescence signal allows quantitative measurements of the accumulation of the PCR-product in the exponential stages to be made [18].‬‬

The set of universal primers for the amplification of *S. scabiei*, SSUDF and SSUDR (generating 135 bp amplicons), was used with a newly designed species-specific TaqMan probe for the identification of *S. scabiei* (ProSc: 5′-GGTAACTTGTATGAAGGGACTAACTAAA-3′).

The probe was designed using Primer 3 (v. 0.4.0) [17]. The TaqMan probe was labelled with a BHQ1 quencher dye (Eurofins Genomics) at 3′-end, and with FAM reporter dye at 5′-end. Amplification reactions contained 0.4 μM of each primer (SSUDF and SSUDR), 0.25 μM of probe (ProSc), 1× Master Mix (TaqMan Universal Master Mix, Applied Biosystems by Life Technologies), 1.5 μL of DNA solution (replaced by water in No Template Controls) and nuclease-free water in a final volume of 15 μL. Cycling conditions for the PCR consisted of a 10-min start-up denaturation step at 95 °C, followed by 45 cycles of amplification for 15 s at 95 °C and 1 min at 60 °C.

### Specificity and technical sensitivity of the conventional end-point PCR universal diagnosis method

The specificity of the generic primers for the universal diagnosis of *S. scabiei* infection was evaluated using reference samples of *S. scabiei* preserved in the authors’ mite collection, as well as heterologous samples of *Psoroptes cuniculi* and *Notoedres cati* var*. cuniculi* collected from tame rabbits *(Oryctolagus cuniculi)*, and *Otodectes cynotis* collected from a dog (*Canis lupus familiaris*). *Psoroptes cuniculi*, *Notoedres cati* var. *cuniculi* and *Otodectes cynotis* were chosen since they are phylogenetically close to *Sarcoptes* mites and are not difficult to obtain. DNA samples extracted from skin biopsies of unexposed badgers (*Meles meles*) and roe deer (*Capreolus capreolus*) were used as negative controls. Specificity was verified by comparing with these negative control samples and by the DNA sequencing of the PCR products.

The sensitivity of our assay was assessed using a twofold dilution series (between 5 ng/μL and 0.01 ng/μL) of *S. scabiei* gDNA. The detection limit was based on the final dilution at which the amplified 135 bp band was still visible in the agarose gel.

### Specificity and technical sensitivity of the TaqMan real-time PCR universal diagnosis method

The specificity of the TaqMan real-time PCR diagnosis method was tested with the same samples as used for testing the specificity of the conventional end-point PCR diagnosis method and with the same criteria.

The sensitivity of our assay was assessed using a twofold dilution series (between 5 ng/μL and 0.0005 ng/μL) of *S. scabiei* gDNA. The limit of detection was based on the final dilution at which the signal of the TaqMan probes was still exponentially amplified.

## Results and discussion

**Fig. 1:**
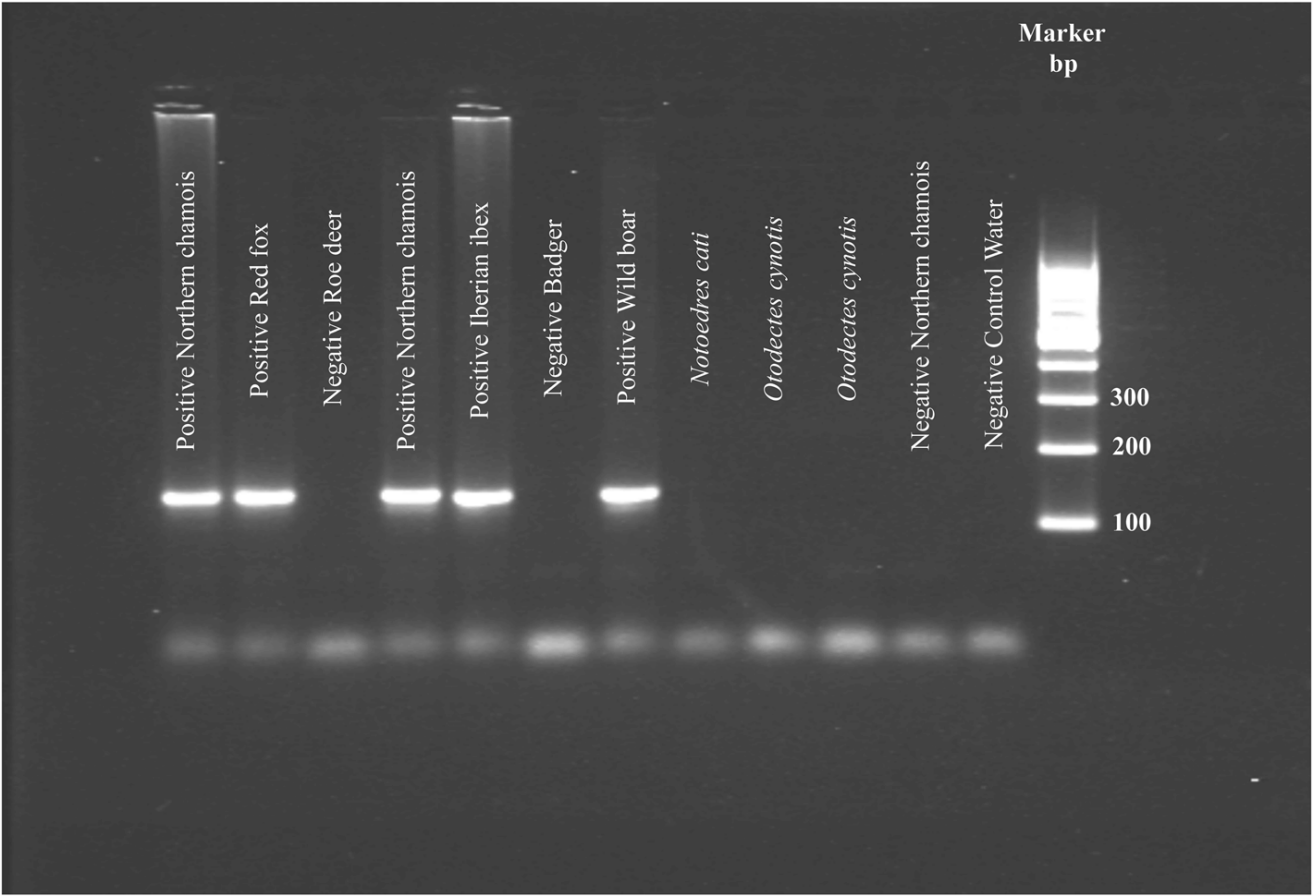
Negative agarose gel showing representative amplicons using the SSUDF and SSUDR primers for the normal end-point PCR universal diagnosis of *Sarcoptes scabiei*

We obtained positive diagnoses for all samples from mangy animals with both diagnosis methods (conventional end-point PCR and TaqMan real-time PCR). No false positives were generated by either test for the heterologous samples from *P. ovis, O. cynotis* and *N. cati*, for the healthy badger and roe deer DNA samples, or for skin scrapings from healthy unexposed animals (Fig. 1).

**Fig. 2:**
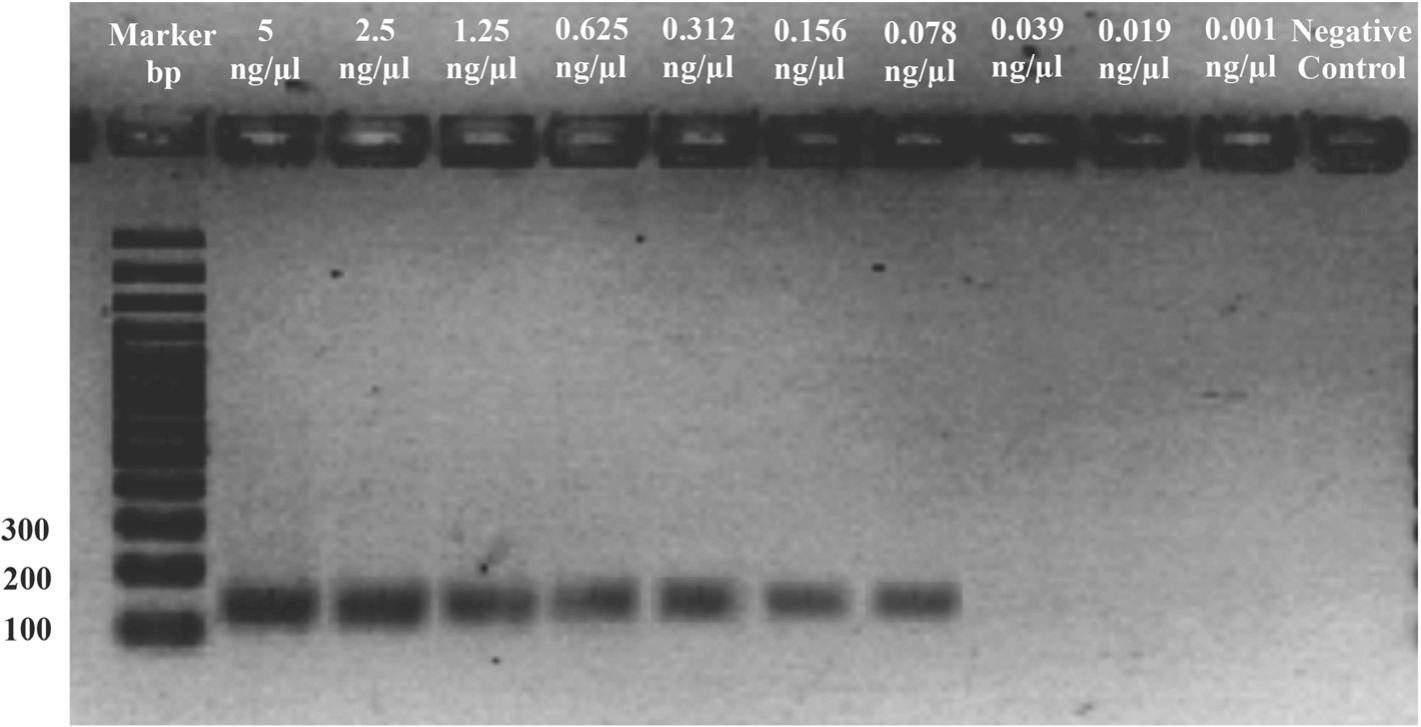
Universal normal end-point PCR amplification of mitochondrial DNA from *Sarcoptes scabiei* (in mangy wolves from Spain) at several dilutions, using the universal primers

**Fig. 3:**
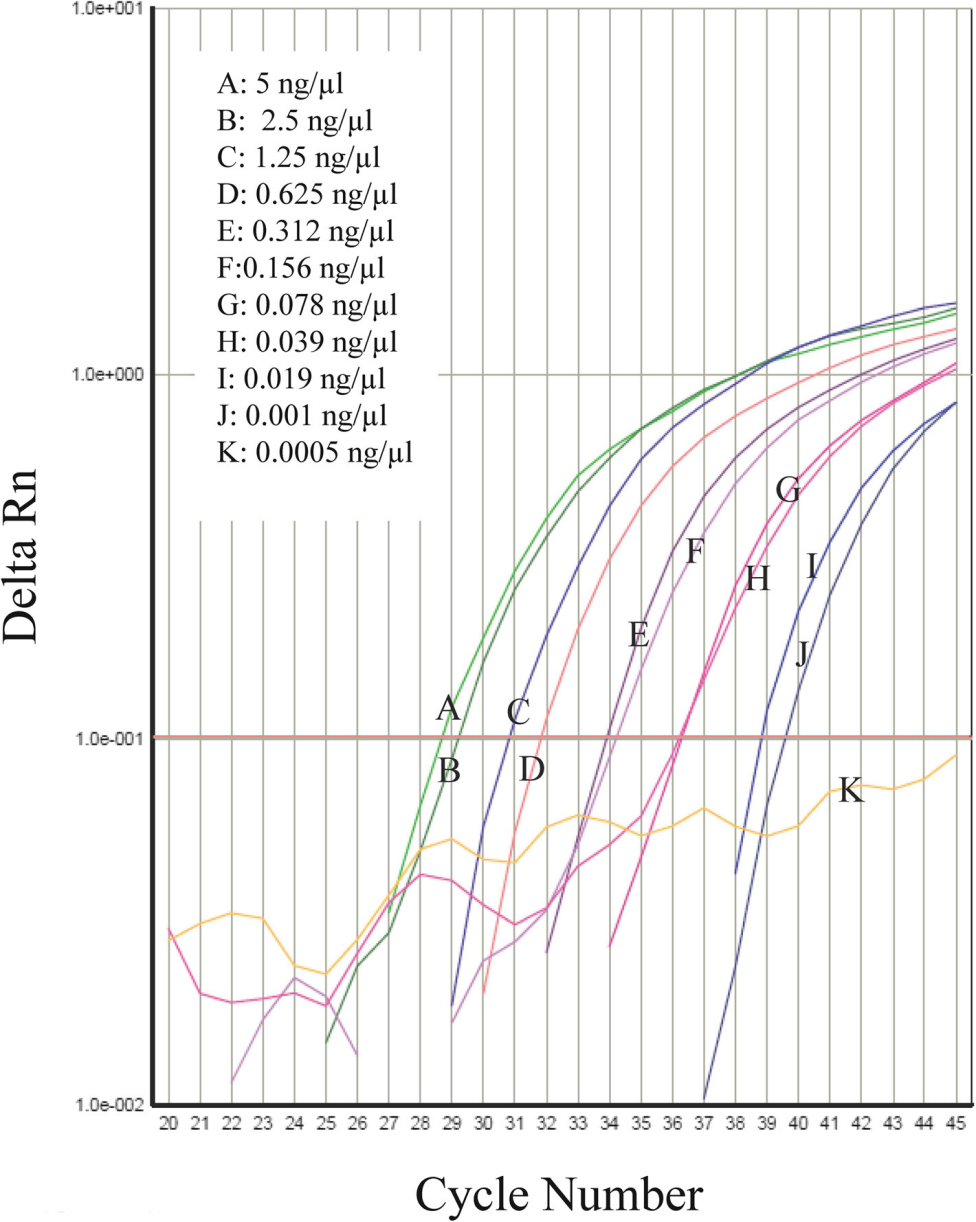
TaqMan RT qPCR amplification of mitochondrial DNA from *Sarcoptes scabiei* at several dilutions

The technical sensitivity of the end-point PCR diagnosis was lower than that of the TaqMan PCR diagnosis. The minimum amount of *Sarcoptes* gDNA detected with conventional end-point PCR was about 80 pg/μL (Fig. 2), whereas only 10 pg/μL was needed for the TaqMan PCR technique (Fig. 3). The higher sensitivity of the TaqMan real-time PCR diagnosis method was expected and can be attributed to the fact that the detection limit in a conventional end-point PCR is based on the final dilution at which a PCR product is still visible in agarose gels, while the fluorophore signal in the TaqMan probes is still detectable at much lower concentrations. The PCR mixtures/conditions of the TaqMan PCR and conventional end-point PCR may also have contributed to this difference.

**Fig. 4:**
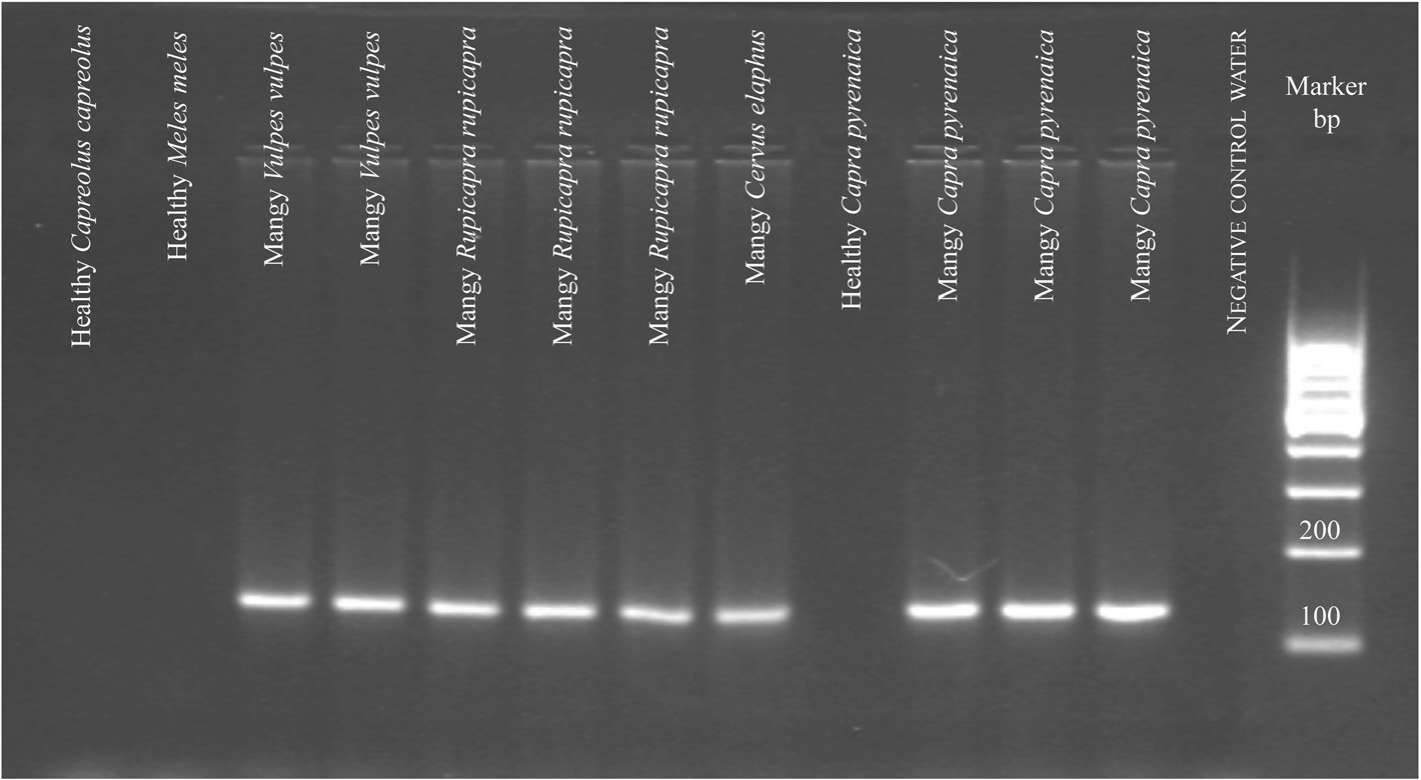
Negative agarose gel showing the results of applying the normal end-point PCR universal diagnosis method for *Sarcoptes scabiei* to skin scrapings from different animal species with varying clinical degrees of sarcoptic mange

Both diagnostic methods were successfully applied to all of the 48 skin scrapings obtained from the six host species. We obtained nine negative results (no amplicons) for skin scrapings from healthy animals and 39 positive results (presence of the amplicons) for the skin scrapings from mangy animals (Fig. 4).

Even given the known advantages of the TaqMan PCR over the end-point PCR [19], This latter diagnosis method is still a good alternative option, above all because the majority of scabies/mange infections occur in countries with poor economic resources where the equipment, materials and expertise needed for the TaqMan qPCR diagnosis method may be lacking.

Additional experimental steps are clearly needed to test how the new methods may contribute to fine-tuning current knowledge of the epidemiology of sarcoptic mange in domestic and wild animals, and of scabies in man, including further studies to test the true sensitivity of the methods, using suspected cases and a gold standard method (or a combination of methods, such as the burrow ink test and handheld dermatoscopy in the case of scabies) as reference [20, 21]. We foresee that, beyond the clinical diagnostic and therapeutic context, the potentialities disclosed by the two methods may find promising application in experiments aimed to investigate the mechanisms of resistance/immunity to infection by *S. scabiei*, including spontaneous recovery in naïve and previously exposed individuals/populations, and the subclinical carrier state of *S. scabiei* in livestock and wildlife, amongst other topics.

## Conclusions

We successfully designed and applied two universal PCR-based diagnosis methods for *S. scabiei*, one based on conventional end-point PCR and the other on TaqMan real-time PCR. These new methods were standardized and found to have high specificity and technical sensibility in 23 host species from 14 counties. They successfully diagnosed (based on skin scrapings) different clinical degrees of sarcoptic mange affecting several animal species. We recommend further testing and the application of these new universal methods worldwide.

## References

[1] Alasaad S, Walton S, Rossi L, Bornstein S, Abu-Madi M (2010). *Sarcoptes*-World Molecular Network (*Sarcoptes*-WMN): integrating research on *scabies*. Int J Infect Dis.

[2] Engelman D, Kiang K, Chosidow O, McCarthy J, Fuller C, Lammie P, Hay R, Steer A (2013). Toward the global control of human scabies: Introducing the International Alliance for the Control of Scabies. PLoS Negl Trop Dis.

[3] Alasaad S, Rossi L, Heukelbach J, Pérez JM, Hamarsheh O, Otiende M, Xing-Quan Z (2013). The neglected navigating web of the incomprehensibly emerging and re-emerging *Sarcoptes* mite. Infect Genet Evol.

[4] Heukelbach J, Wilcke T, Winter B, Feldmeier H (2005). Epidemiology and morbidity of scabies and pediculosis capitis in resource-poor communities in Brazil. Br J Dermatol.

[5] Heukelbach J, Feldmeier H (2006). Scabies. Lancet.

[6] Walton SF, Currie BJ (2007). Problems in Diagnosing Scabies, a Global Disease in Human and Animal Populations. Clin Microbiol Rev.

[7] Alasaad S, Permunian R, Gakuya F, Mutinda M, Soriguer RC, Rossi L (2010). Sarcoptic-mange detector dogs used to identify infected animals during outbreaks in wildlife. BMC Vet Res.

[8] Alasaad S, Sarasa M, Heukelbach J, Mijele D, Soriguer RC, Rossi L (2014). Advances in studies of disease-navigating webs: *Sarcoptes scabiei* as a case study. Parasit Vectors.

[9] Jacobson M, Bornstein S, Wallgren P (1997). The efficacy of simplified eradication strategies against sarcoptic mange mite infections in swine herds monitored by an ELISA. Vet Parasitol.

[10] Jacobson M, Bornstein S, Palmer E, Wallgren P (2000). Elimination of *Sarcoptes scabiei* in pig herds by single and double administrations of an avermectin. Acta Vet Scand.

[11] Graczyk TK, Mudakikwa AB, Cranfield MR, Eilenberger U (2001). Hyperkeratotic mange caused by *Sarcoptes scabiei* (Acariformes: Sarcoptidae) in juvenile human-habituated mountain gorillas (*Gorilla gorillaberingei*). Parasitol Res.

[12] Alasaad S, Rossi L, Soriguer RC, Rambozzi L, Soglia D, Pérez JM (2009). *Sarcoptes* mite from collection to DNA extraction: the lost realm of the neglected parasite. Parasitol Res.

[13] Alasaad S, Rossi L, Maione S, Sartore S, Soriguer RC, Pérez JM (2008). HotSHOT Plus ThermalSHOCK, a new and efficient technique for preparation of PCR-quality *Sarcoptes* mite genomic DNA. Parasitol Res.

[14] Soglia D, Rambozzi L, Maione S, Spalenza V, Sartore S, Alasaad S, Sacchi P, Rossi L (2009). Two simple techniques for the safe *Sarcoptes* collection and individual mite DNA extraction. Parasitol Res.

[15] Alasaad S, Soglia D, Maione S, Sartore S, Soriguer RC, Pérez JM, Rasero R, Rossi L (2009). Effectiveness of the postponed isolation (post-frozen isolation) method for PCR-quality *Sarcoptes* mite gDNA. Exp Appl Acarol.

[16] Hall TA (1999). BioEdit: a user friendly biological sequence alignment editor and analysis program for Windows 95/98/NT. Nucleic Acids Symp Ser.

[17] Rozen S, Skaletsky HJ, Krawetz S, Misener S (2000). Primer3 on the WWW for general users and for biologist programmers. Bioinformatics methods and protocols: methods in molecular biology.

[18] Holland PM, Abramson RD, Watson R, Gelfand DH (1991). Detection of specific polymerase chain reaction product by utilizing the 5′----3′ exonuclease activity of *Thermus aquaticus* DNA polymerase. Proc Natl Acad Sci U S A.

[19] VanGuilder HD, Vrana KE, Freeman WM (2008). Twenty-five years of quantitative PCR for gene expression analysis. BioTechniques.

[20] Verweij JJ, Stensvold CR (2014). Molecular testing for clinical diagnosis and epidemiological investigations of intestinal parasitic infections. Clin Microbiol Rev.

[21] Leung V, Miller M (2011). Detection of scabies: A systematic review of diagnostic methods. Can J Infect Dis Med Microbiol.

