# Erratum: Universal conventional and real-time PCR diagnosis tools for *Sarcoptes scabiei*

**DOI:** 10.1186/s13071-015-1238-y

**Published:** 2015-12-07

**Authors:** Samer Angelone-Alasaad, AnnaRita Molinar Min, Mario Pasquetti, Abdulaziz N. Alagaili, Stefano D’Amelio, Federica Berrilli, Vincent Obanda, Mohamed A. Gebely, Ramón C. Soriguer, Luca Rossi

**Affiliations:** Dipartimento di Scienze Veterinarie, Largo Paolo Braccini 2, 10095 Grugliasco, Italy; Institute of Evolutionary Biology and Environmental Studies (IEU), University of Zürich, Winterthurerstrasse 190, 8057 Zürich, Switzerland; Department of Zoology, College of Science, King Saud University, Riyadh, Saudi Arabia; Department of Public Health and Infectious Diseases, University of Rome La Sapienza, Roma, Italy; Department of Experimental Medicine and Surgery, University of Rome Tor Vergata, Roma, Italy; Department of Veterinary Services, Kenya Wildlife Service, Nairobi, Kenya; Department of Parasitology and Animal Diseases, Veterinary Research Division, National Research Center, Dokki, Giza, Egypt; Estación Biológica de Doñana, Consejo Superior de Investigaciones Científicas (CSIC), Avda. Américo Vespucio s/n 41092, Sevilla, Spain

## Erratum

After the publication of this work [1], we noticed an error in the Methods Section. The reported sequence of the ProSc “5′-GGTAACTTGTATGAAGGGACTAACTAAA-3′” is not the correct one. The correct one is “ProSc: 5′-GGTAACTTGTATGAAGGRAYTAACTAAA-3′”.
